# 3D-Printed Coronary Plaques to Simulate High Calcification in the Coronary Arteries for Investigation of Blooming Artifacts

**DOI:** 10.3390/biom11091307

**Published:** 2021-09-03

**Authors:** Zhonghua Sun, Curtise Kin Cheung Ng, Yin How Wong, Chai Hong Yeong

**Affiliations:** 1 Discipline of Medical Radiation Science, Curtin Medical School, Curtin University, Perth, WA 6845, Australia; curtise.ng@curtin.edu.au; 2 Curtin Health Innovation Research Institute (CHIRI), Faculty of Health Sciences, Curtin University, Perth, WA 6845, Australia; 3 Faculty of Health & Medical Sciences, School of Medicine, Taylor’s University, No. 1, Jalan Taylor’s, Subang Jaya 47500, Malaysia; yinhow.wong@taylors.edu.my (Y.H.W.); chaihong.yeong@taylors.edu.my (C.H.Y.)

**Keywords:** 3D printing, coronary artery disease, model, accuracy, calcification, plaque

## Abstract

The diagnostic value of coronary computed tomography angiography (CCTA) is significantly affected by high calcification in the coronary arteries owing to blooming artifacts limiting its accuracy in assessing the calcified plaques. This study aimed to simulate highly calcified plaques in 3D-printed coronary models. A combination of silicone + 32.8% calcium carbonate was found to produce 800 HU, representing extensive calcification. Six patient-specific coronary artery models were printed using the photosensitive polyurethane resin and a total of 22 calcified plaques with diameters ranging from 1 to 4 mm were inserted into different segments of these 3D-printed coronary models. The coronary models were scanned on a 192-slice CT scanner with 70 kV, pitch of 1.4, and slice thickness of 1 mm. Plaque attenuation was measured between 1100 and 1400 HU. Both maximum-intensity projection (MIP) and volume rendering (VR) images (wide and narrow window widths) were generated for measuring the diameters of these calcified plaques. An overestimation of plaque diameters was noticed on both MIP and VR images, with measurements on the MIP images close to those of the actual plaque sizes (<10% deviation), and a large measurement discrepancy observed on the VR images (up to 50% overestimation). This study proves the feasibility of simulating extensive calcification in coronary arteries using a 3D printing technique to develop calcified plaques and generate 3D-printed coronary models.

## 1. Introduction

Coronary computed tomography angiography (CCTA) is a widely used imaging modality in the diagnostic assessment of coronary artery disease (CAD) owing to advanced computed tomography (CT) scanning techniques that allow for the acquisition of high resolution imaging data [1,2,3]. However, the main challenge of CCTA lies in the assessment of heavily calcified plaques in the coronary arteries because extensive calcification produces blooming artifacts that result in high false positive rates, thus significantly reducing the specificity and positive predictive value (PPV) [4,5,6,7]. To address this issue, different strategies have been proposed and these include uses of image post-processing algorithms, dual-energy CT, iterative reconstruction (IR), and so on for suppressing the blooming artifacts with improved specificity and PPV, to some extent [8,9,10,11,12]. A recently developed vendor-specific deblooming algorithm has been shown to improve the diagnostic performance of CCTA, with specificity and PPV increased from 45.8% and 69.8% to 75% and 83.3%, respectively. Despite promising findings from this new algorithm, it is limited to a specific vendor. Further, the cardiac phantom used in their study does not represent realistic coronary arteries, which is another limitation [13].

In this study, we aimed to tackle this challenge by utilising a three-dimensional (3D) printing or additive manufacturing technique for the simulation of calcification in patient-specific coronary artery models. Over the last decade, there have been increased applications of 3D printing in cardiovascular disease, with the main clinical value of 3D-printed models focusing on presurgical planning and simulation of complex cardiac surgeries, education of medical students and healthcare professionals, and improving doctor–patient communication [14,15,16,17,18,19,20,21,22,23,24]. Another emerging research area in 3D printing in medicine includes the study of optimal CT scanning protocols using 3D-printed anatomical phantoms. Our previous reports have shown the feasibility of simulating coronary plaques and coronary stents in personalised coronary models [17,25,26,27]. The present study was conducted to further advance the applications of 3D printing in the study of calcified plaques, and it was performed in two stages. Stage 1 involved the simulation of calcification in the coronary arteries by creating plaque models in different diameters with CT attenuation similar to high calcification, followed by insertion of these plaques into the 3D-printed patient-specific coronary artery models to simulate different degrees of coronary stenosis. Stage 2 included CT scanning of the 3D-printed coronary models and reconstruction of two-dimensional (2D) and 3D images for visualisation of these calcified plaques and measurement of the plaque diameters to compare them with the actual sizes for determination of the blooming artifacts of high calcification and their impact on the measurement accuracy between 2D and 3D images. It is expected that the findings of this study will lay foundation for further investigation of the blooming artifacts associated with severe coronary calcification, thus developing strategies for improving the diagnostic performance of CCTA.

## 2. Materials and Methods

### 2.1. Generation of 3D Coronary Artery and Plaque Models for 3D Printing

The creation of patient-specific 3D-printed coronary artery models has been described in previous studies [26,27]. In brief, anonymised CCTA images in DICOM (digital imaging and communications in medicine) were selected for image post-processing and segmentation with only coronary arteries extracted from the volume data. In this study, six CCTA datasets were selected for generation of a 3D coronary artery tree, which consisted of the main coronary branches, including the right coronary artery (RCA) as well as left anterior descending (LAD) and left circumflex (LCx) arteries in each case.

**Figure 1:**
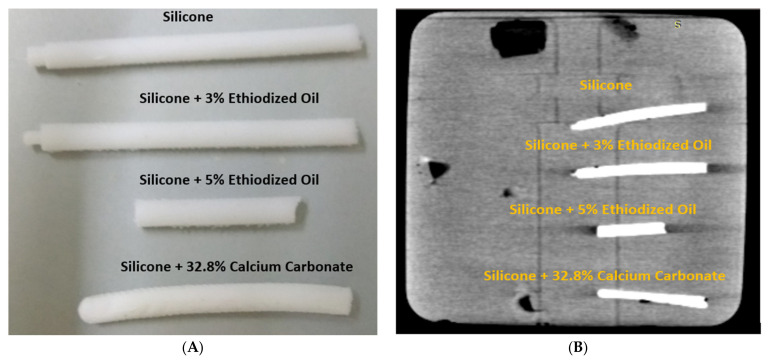
Creation of simulated calcified plaques. (**A**) Material compositions to simulate calcification in the 3D-printed mould. (**B**) CT image of these materials.

The approach to simulate calcification was employed through testing mixtures of different materials. We tested the following four compositions to determine the appropriate material with CT attenuation close to 800 HU (Hounsfield unit): silicone, silicone + 3% ethiodized oil, silicone + 5% ethiodized oil, and silicone + 32.8% calcium carbonate. The simulated calcified plaques were produced by the moulding technique [28]. The mould (circular rod) was first printed with polylactic acid (PLA) using a 3D printer (Ultimaker 2 + Extended, Ultimaker BV, Geldermalsen, The Netherlands). Then, the composition materials to resemble the calcified plaques were injected into the mould, forming the plaques. Figure 1A shows the simulated plaques with use of these materials, while Figure 1B shows the CT image (with 120 kV) of these plaques with CT attenuation measured: 450 HU, 600 HU, 900 HU, and 800 HU, respectively, corresponding to these compositions. It is well known that a coronary calcium score of above 400 is used as a reliable indicator to predict cardiovascular events, and the selection of 800 HU for calcified plaques is based on findings from the study of Bischoff et al., which showed the mean HU threshold for CCTA calcium scoring was 672 HU in 120 kV scanning based on an analysis of 500 patients with suspected coronary artery disease [29]. Hence, the combination of silicone and 32.8% calcium carbonate was chosen as the material for simulating calcified plaques.

### 2.2. 3D-Printed Coronary Artery for Simulation of Coronary Calcification

**Figure 2:**
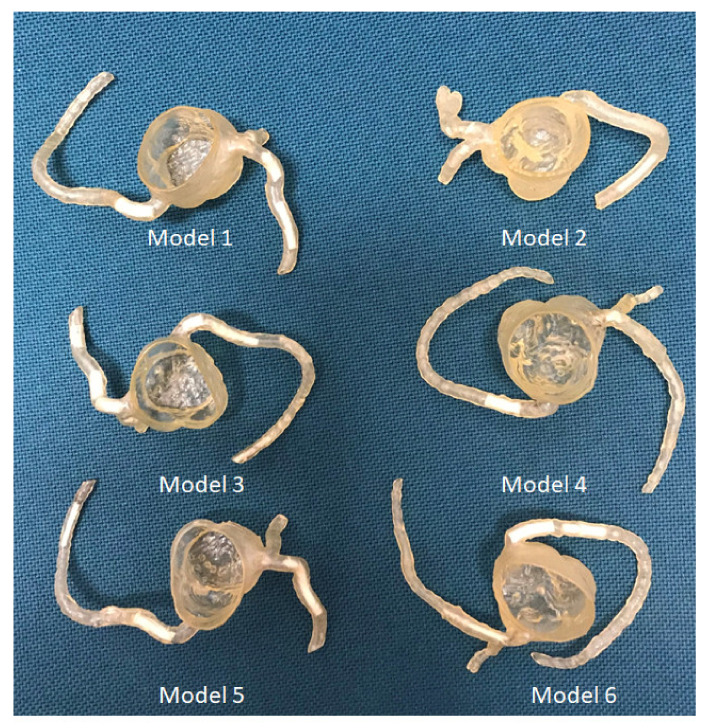
3D-printed coronary artery models with simulated calcified plaques inserted into the coronary artery branches.

The coronary artery model was printed using a digital light processing (DLP) 3D printer (Photon S, Anycubic, Shenzhen, Guangdong, China) with printing resolution of 50 µm for the X and Y axis planes. The resolution for the z-axis could be manipulated from 25 µm to 100 µm. The 3D models were printed with 50 µm resolution at the z-axis in this study, thus the printing resolution was isotropic. The material used was the photosensitive polyurethane (PU) resin with a shore hardness of 80A (Fabbxible Technology Sdn Bhd, Malaysia). These calcified plaques were inserted into different locations at the coronary artery branches to simulate heavy calcification. Although the 3D-printed plaques had high CT attenuation, simulating heavy calcification, they were quite soft and elastic, and hence did not cause any distortion to the coronary wall after being inserted into the arteries. A total of 22 plaques with different diameters and lengths were placed at different segments (from proximal to distal) of these six coronary models, as shown in Figure 2. Plaque details and locations in coronary arteries are summarized in Table 1. The diameter of these calcified plaques ranges from 1 mm to 4 mm, simulating different degrees of coronary stenosis, ranging from 20% to 80%.

Printing of these models took around 5 to 10 h. We only carried out regular washing to remove the unpolymerized resin material and removed the support material after the printing. Other than that, we did not apply any other post-processing to the print result to ensure the accuracy and reliability of the 3D-printed models.

### 2.3. Coronary CT Scanning Protocols

**Figure 3:**
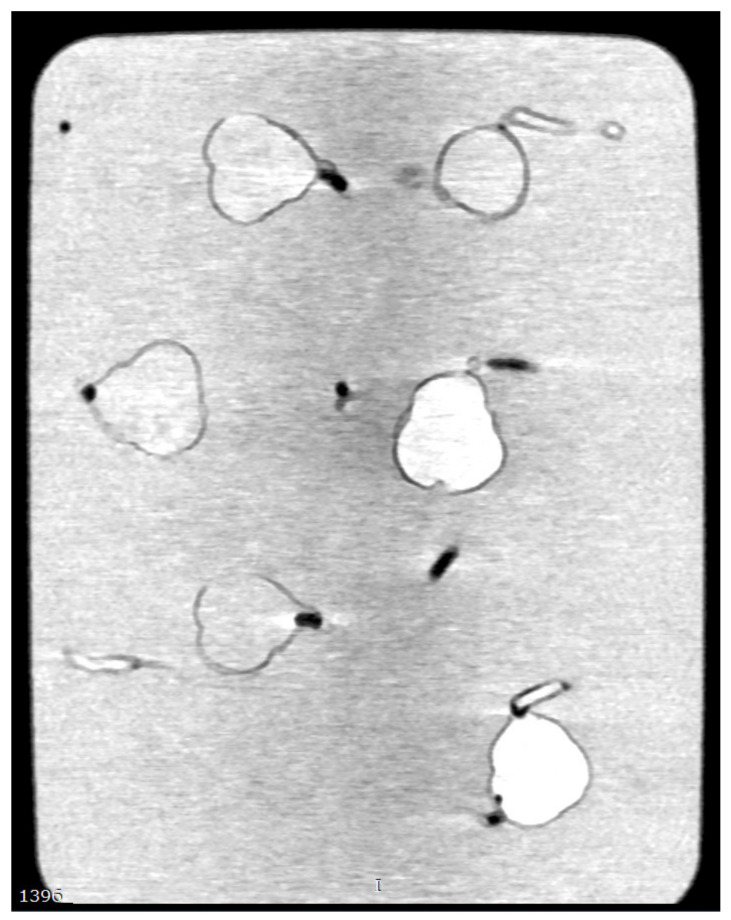
2D multiplanar coronal reformation shows coronary artery models with contrast medium filling the models and the container. Calcified plaques are difficult to be differentiated from the high attenuation background.

**Figure 4:**
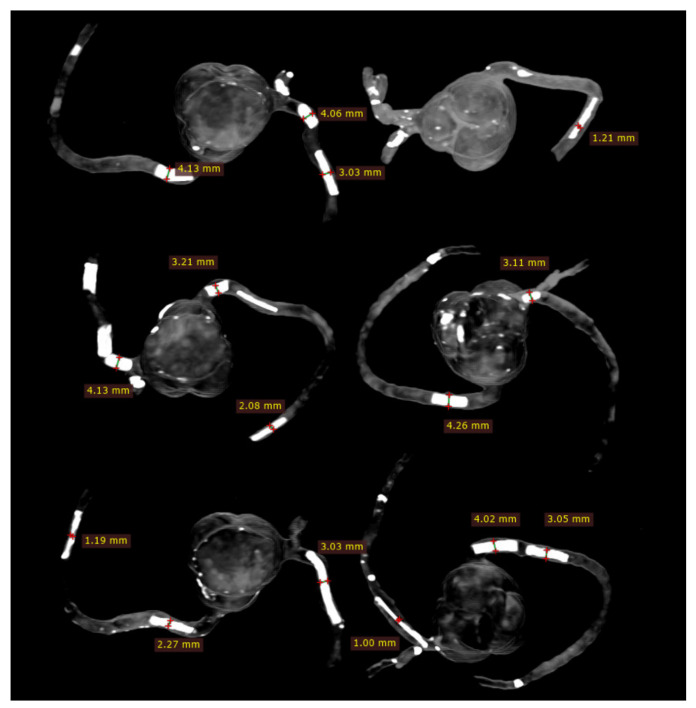
Measurements of plaque dimensions on 2D maximum-intensity projection images using 0.5 mm slice thickness.

**Figure 5:**
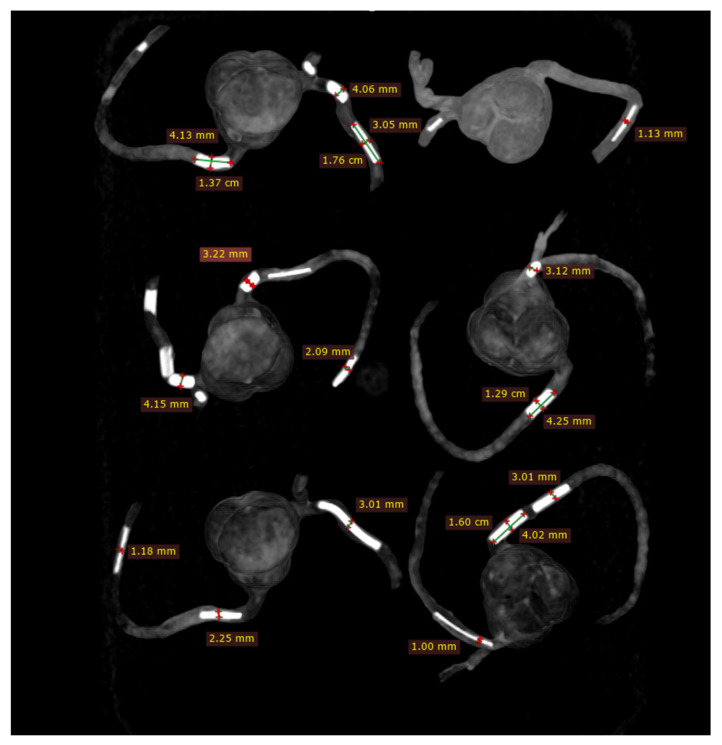
Measurements of plaque dimensions on 2D maximum-intensity projection images with 1.0 mm slice thickness.

CT scan was performed on a 192-slice dual-source CT scanner (Siemens Force, Siemens Healthcare, Forchheim, Germany) with these coronary models placed in a plastic container. Electrocardiographic gating was not used because of the static nature of these models, and the following protocol was used: slice thickness of 1.0 mm with a 0.5 mm reconstruction interval; gantry rotation time of 0.25 s; tube potential and current of 70 kV and 20 mA, respectively; and pitch of 1.4. We first tested CT scans with contrast medium Omnipaque TM350 (diluted to 7% simulating CT attenuation of 300 HU) filling the 3D printed coronary models and the container; however, it was difficult to visualise the calcified plaques because of overlapping between contrast medium inside and outside the coronary arteries and the plaques, as shown in Figure 3. Thus, we decided to scan the models without contrast medium to allow a clear demonstration of simulated calcification in the coronary arteries for dimensional measurements. Despite the standard cardiac CT angiography using 0.5 mm slice thickness, we did not observe any significant difference in measuring plaque dimensions when compared with those measured on images acquired with 1.0 mm slice thickness (*p* > 0.05). Figure 4 shows the measurements of plaque diameters on maximum-intensity projection (MIP) images acquired with 0.5 mm slice thickness and 70 kV, similar to those on MIP images acquired with 1.0 mm slice thickness and 70 kV (Figure 5). Table 2 shows a comparison of the diameter measurements between images acquired with 0.5 and 1.0 mm slice thicknesses in these plaques, as demonstrated in Figure 4 and Figure 5. Thus, we chose to scan the 3D-printed models with use of 1.0 mm slice thickness and 70 kV. The reason for using 70 kV was to place these models in a small container instead of a realistic chest phantom. Further, low kV allowed for maximal visualisation of high attenuation calcification compared with high kV, owing to the k edge effect of high calcification at the low photon energy scans. In this study, the CT attenuation of calcified plaques was measured to be around 1200 HU at 70 kV, as opposed to 800 HU measured at 120 kV, and this allowed us to study the blooming artifacts associated with extensive calcification in the coronary arteries.

### 2.4. 2D and 3D Image Reconstruction and Assessment

**Figure 6:**
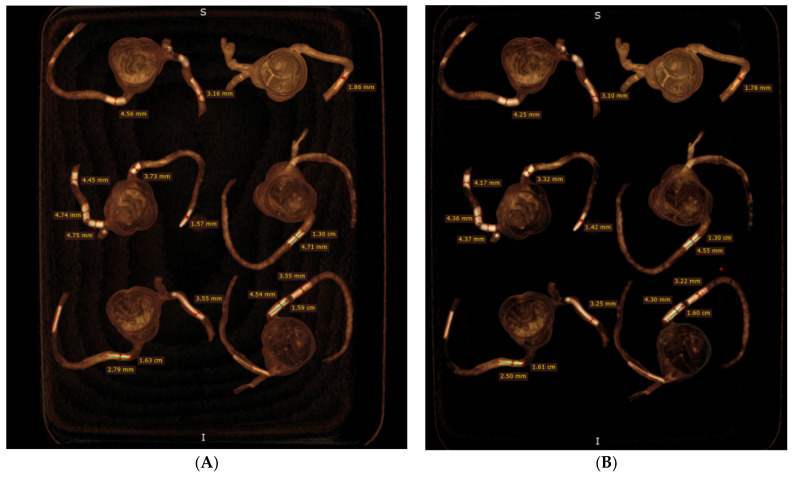
Measurement of plaque dimensions on 3D volume rendering images with the use of different windowing. (**A**) Measurements of plaque dimensions on wide window width (window width and window level: 1250 and 250). (**B**) Measurement of plaque dimensions at the same plaque locations with narrow window width (window width and window level: 1050 and 210).

CCTA images in DICOM format were transferred to a separate workstation for image processing and analysis using RadiAnt DICOM viewer 2020.1.1 (Medixant, Poznan, Poland). Two-dimensional axial images were reconstructed into MIP and 3D volume rendering (VR) views for visualisation of calcified plaques and measurements of plaque dimensions. For VR views, we first used a wide window width (window width and window level: 1250 and 250) to allow visualisation of plaque and coronary lumen. Then, we narrowed the window width (window width and window level: 1050 and 210) to focus on visualisation of the calcified plaques. Measurements of plaque diameter and length were performed on MIP and VR images (wide and narrow window widths), with the results compared to those from the actual sizes of these plaques and any significant differences determined. Measurements were conducted by an assessor (with more than 15 years of experience in cardiac CT imaging) and performed three times at different locations of each plaque with the mean values used as the final ones for comparisons. Figure 5 shows an example of measuring these plaques based on the MIP images, while Figure 6 displays measurements based on two VR views with the use of wide and narrow window widths. According to Society of Cardiovascular Computed Tomography (SCCT) guidelines, although multiplanar reformation is preferred to delineate the plaque morphology and coronary lumen changes, MIP images are useful for identifying artifacts, as well as the presence and position of the coronary lesions, while VR images are useful for demonstrating spatial relationships between coronary lesions and vessels [30]. As our purpose of this study was to focus on the blooming artifacts associated with heavy calcifications in the coronary arteries instead of assessing lumen stenosis, we compared MIP with VR visualisation tools in this experiment.

### 2.5. Statistical Analysis

SPSS version 26.0 (IBM, Armonk, NY, USA) was used for the statistical analyses. Quantitative variables were displayed as mean and standard deviation. Three-way analysis of variance (ANOVA) with pairwise comparisons post-hoc tests were performed to assess any significant differences between mean measurements on 2D MIP and VR (with wide and narrow window width) views with the original measurements as the reference. A *p*-value <0.05 indicated statistical significance.

## 3. Results

**Figure 7:**
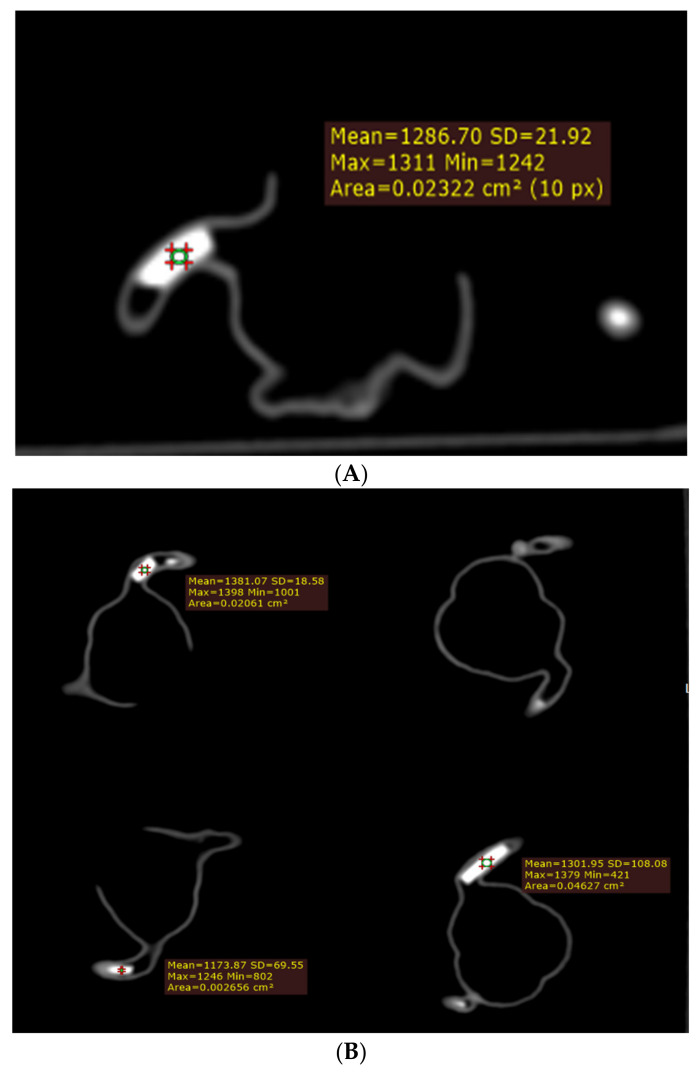

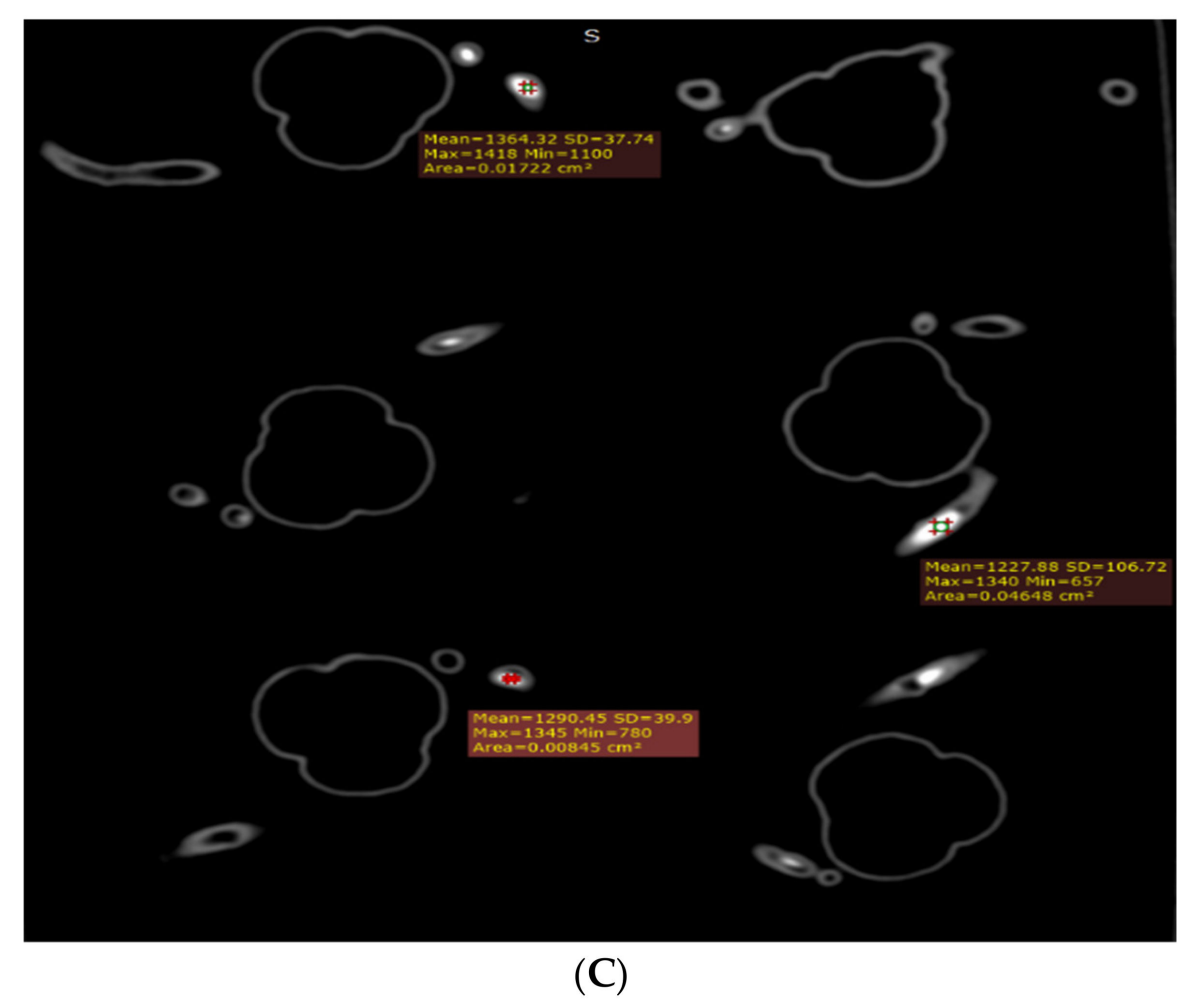
Measurement of CT attenuation at the simulated calcified plaques on 2D axial images (**A**–**C**).

The mean plaque diameters and lengths measured with MIP and VR are presented in Table 3. These simulated calcified plaques are successfully demonstrated in these coronary artery branches, showing high CT attenuation. The CT attenuation of these plaques was measured to range from 1100 to nearly 1400 HU, as shown in Figure 7. The higher attenuation than the originally tested 800 HU on these plaques is because of the k edge at the calcium carbonate material at low kV scanning, contributing to high attenuation.

Plaque diameters were all overestimated on MIP and VR images with physical measurements of these plaques used as the reference values (Table 1); however, measurements on MIP images were close to the actual diameters in all plaques, with less than 10% accuracy difference in the plaques with a diameter between 2 and 4 mm (Table 3). In five calcified plaques with 1 mm diameter, more than 10% (12–23%) measurement difference was noticed in three of them on MIP images. In contrast, both VR views overestimated the plaque diameters by more than 10% in most of these plaques, with the highest overestimation reaching 50% in plaque 19 (model 5), as shown in Table 3. Although the mean measurement differences in plaque diameters were slightly reduced when narrow windowing was applied to VR images as opposed to the VR views with wide window width showing both coronary lumen and plaques, there were no significant differences in the measurement accuracy between these two VR views (*p* > 0.05). Significant differences were found between MIP and two VR views in measuring all of the plaque diameters (*p* < 0.05) (Table 3). There was no significant difference in measuring the plaque lengths between MIP and two VR views, regardless of the plaque sizes and locations (*p* > 0.05) (Table 4).

**Figure 8:**
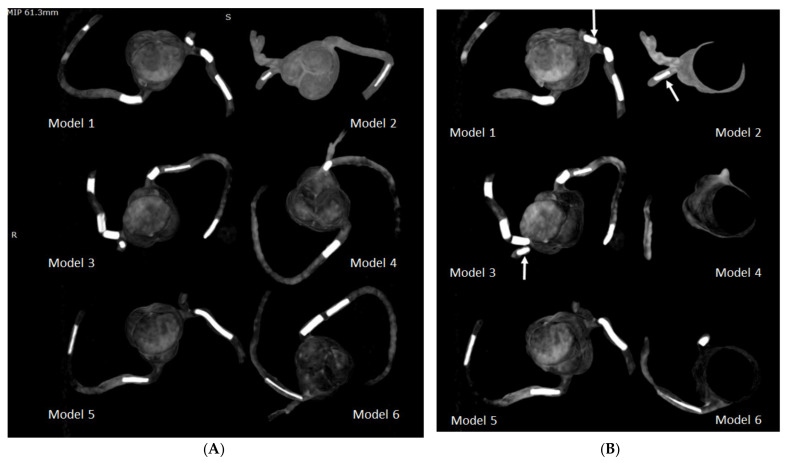
Maximum-intensity projection (MIP) images showing the calcified plaques in six 3D-printed coronary models. (**A**) Coronal MIP view showing these calcified plaques. (**B**) Oblique MIP view showing the plaques more clearly in the left circumflex coronary artery (arrows) in model 1 (plaque 3), model 2 (plaque 6), and model 3 (plaque 11).

**Figure 9:**
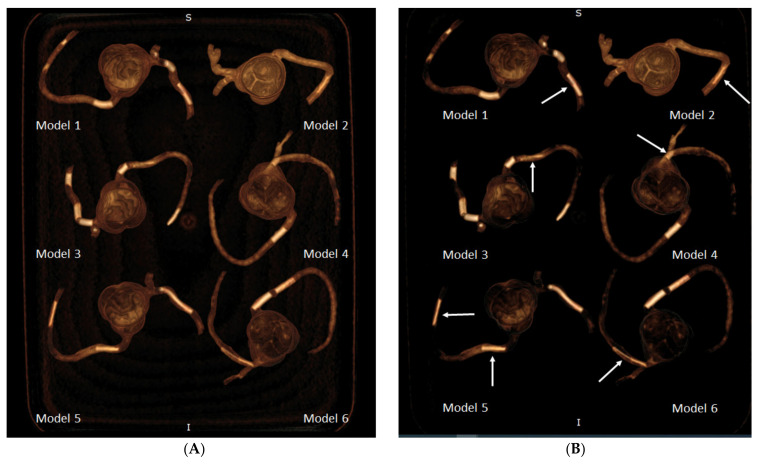
3D volume rendering (VR) views showing calcified plaques in the coronary arteries. (**A**) VR views with wide window width (window width and window level: 1250 and 250) showing both plaques and coronary lumen in these 3D-printed models. (**B**) When narrow window width (window width and window level: 1050 and 210) is applied, high attenuation plaques are visualised more clearly, with some of the coronary lumen not displayed. This is especially apparent when visualising model 1 (plaque 2), model 2 (plaque 7), model 3 (plaque 13), model 4 (plaque 15), model 5 (plaques 18 and 19), and model 6 (plaque 20). Arrows refer to these plaques better visualised on VR images with narrow window width.

Figure 8 demonstrates coronal and oblique MIP images showing these calcified plaques in the coronary artery models, while Figure 9 presents VR images with window width changed. These 2D and 3D images clearly demonstrate the calcified plaques in the coronary arteries.

## 4. Discussion

In the present study, we simulated highly calcified coronary plaques and tested these plaques in the 3D-printed coronary artery models. The results showed overestimation of plaque diameters on both MIP and VR views owing to the blooming artifacts, with the measurements based on the MIP images close to the actual plaque sizes. A significant difference was found in the measurement of plaque diameters between the MIP and VR images, with the VR views resulting in greater overestimation of the plaque sizes (up to 50% overestimation). This study extends the application of 3D printing to a challenging area with difficulty in accurately assessing calcified coronary plaques by CCTA, and the preliminary results of this study are promising to encourage further research to address the blooming artifacts associated with the coronary calcification.

Our results are consistent with previous reports confirming the inaccuracy of CCTA in dealing with larger or massive calcifications [5,6,7,8,31]. Despite widespread use of CCTA in the diagnosis of CAD, the presence of coronary calcification produces blooming artifacts interfering with the diagnostic accuracy of CCTA, primarily manifested as the low specificity and PPV. Previous studies have reported that the specificity and PPV of CCTA are less than 50% in patients with highly calcified plaques [4,6,32]. Pontone et al. in their study compared high resolution with standard resolution CCTA (0.23 mm vs. 0.625 mm) in 184 patients at high risk for CAD with invasive coronary angiography as the reference method. Their results showed significant improvements in both specificity and PPV for the high resolution CCTA as opposed to the standard CCTA in both patient-based and segment-based analyses. However, when assessing large or extensive calcifications in the coronary arteries, the agreement between the high resolution CCTA and invasive coronary angiography reduced from 89% to 73%, with 27% of plaques being overestimated [33]. Thus, researchers explored other strategies to suppress the blooming artifacts associated with calcifications in the coronary arteries.

The use of image postprocessing algorithms in highly calcified plaques has addressed this issue to some extent, with promising results reported in the literature [8,9,10,11,12]. These methods include IR, image subtraction, or sharpening algorithms. IR has been shown to significantly reduce the calcium volume and calcium score when compared with filtered back projection, thus increasing specificity and PPV in per-segment and per-patient levels [9,11]. However, contradictory findings were reported by other studies showing no significant impact of using IR on calcium volume change or coronary diameter measurement [34,35,36]. Thus, IR is not recommended to be applied in CCTA images with high calcification. A study by Tanaka et al. used coronary calcium subtraction in CCTA images with high calcium score and their developed subtraction CCTA improved the diagnostic performance of CCTA, but still at a low to moderate value, as the specificity and PPV were increased to 59% and 48% when compared with 48% and 43% with conventional CCTA [37]. Similarly, our previous experience of using postprocessing (sharpening) algorithm increased the specificity and PPV from the 33% and 41% with original CCTA to 66% and 57%, respectively [38]. The deblooming algorithm recently developed by the GE Healthcare was reported to improve CCTA assessment of calcified plaques [13,39], but it was limited to a specific vendor restricting its clinical application. In spite of these efforts, the moderate diagnostic value of CCTA in the assessment of extensive calcifications prompts the need to develop appropriate strategies to address this challenging, but still not satisfactorily resolved issue.

The novelty of our study can be ascribed in two aspects: creation of high calcification for simulation of heavily calcified plaques and use of patient-specific 3D-printed coronary models for simulation of CAD to study blooming artifacts. Three-dimensional printing is showing great promise in the domain of cardiovascular disease [14,15,16,17,18,19,20,21,22,23,24]. In this study, we extended the application of using personalised 3D-printed coronary models to study calcified plaques and associated blooming artifacts. The 3D-printed coronary artery models are advantageous to a simple tube model, which does not replicate realistic coronary anatomy [40,41], while 3D printing allows for creation of individual anatomy and simulation of disease. Three-dimensional-printed personalised aorta, pulmonary, and coronary artery models created from CT angiographic images replicate realistic anatomy and pathology, thus serving as an excellent tool for studying optimal CT scanning protocols [42,43,44,45]. In our previous reports, we simulated pulmonary embolism in the main and side pulmonary artery branches using patient-specific 3D-printed pulmonary artery model and tested different CT pulmonary angiography (CTPA) protocols consisting of a range of kV and pitch values. Optimal CTPA protocols were suggested with kV lowered to 70 or 80 combined with pitch value of 2.2 or 3.2 without significantly affecting image quality, but with a more than 80% reduction in radiation dose [44,45]. In another study, we tested the feasibility of using 3D-printed coronary models for simulation of coronary plaques using high resolution synchrotron radiation CT with regard to the accuracy of determining coronary stenosis [26]. Our recent study on simulation of coronary stenting in 3D-printed coronary models further confirms the advantages of using 3D-printed models to study coronary CT scanning protocols [27]. Stent structures and stented lumen were clearly visualised with images reconstructed with soft and sharp kernels. In the presence of multiple stent wires in tortuous coronary branches, visualisation of stented lumen was significantly affected owing to beam hardening artifacts [27]. These previous reports highlight the promising role of utilising 3D-printed coronary models towards research investigation of CT scanning techniques.

The current study represents the first study that aims to address the gap in the existing literature, and our results confirm the potential application of simulating heavy calcification in the coronary arteries using the 3D printing technique. Three-dimensional- printed coronary models are reported in several studies, primarily focusing on education of understanding complex anatomic structures and in guiding interventional procedures to improve treatment outcomes [46,47,48,49,50]. To the best of our knowledge, no report is available about using 3D-printed coronary models to simulate high calcification. We have achieved the expected results by demonstrating these calcified plaques with variable diameters and lengths in the coronary arteries, based on the 2D MIP and 3D VR images (Figure 5, Figure 6, Figure 8 and Figure 9), and presented the measurement differences in terms of the overestimation of plaque diameters owing to the blooming artifacts. The preliminary findings of this study are considered valuable to the current literature and will encourage more research towards studying highly calcified plaques.

Our aim was to use the realistic coronary models for simulation of high calcification in coronary arteries; however, there are some limitations in this study that should be acknowledged. First, no contrast medium was administered to these 3D-printed coronary models while plaques were in place, thus there was no assessment of coronary lumen change in the presence of high calcification, nor did we apply any post-processing methods to these images. This will be addressed in our further experiments to simulate realistic CCTA with coronary models connected to a cardiac pump simulating heart beats. Second, this study is only a pilot testing of cardiac CT scan on the 3D-printed coronary models with calcified plaques. Hence, we did not perform a series of scans with different protocols as we did previously with the CTPA study [44,45]. The main purpose of this study was to create highly calcified plaques and then simulate calcification in the 3D-printed coronary models. Further, our simulated plaques are homogeneous with circular appearance, while in the clinical situation, the calcified plaques are heterogeneous with different or irregular configurations such as concentric or eccentric appearances, which could result in irregular coronary lumen changes [51]. This needs to be considered in future experiments. Ideally, the coronary CT scans should be performed with the use of standard 0.5 mm slice thickness, and this is going to be investigated in our ongoing study, which will test different coronary CT scanning protocols on the 3D-printed models. The current results have achieved the expected outcomes, thus laying the foundation for further study to focus on the effect of blooming artifacts owing to the high calcification, which will lead to optimal CCTA protocols. Finally, although we successfully simulated calcified plaques in these 3D-printed coronary models, we did not insert the coronary models into a chest phantom with anatomical structures including lungs, chest wall, ribs, thoracic vertebrae, and heart. Future studies should aim to place the coronary models in a realistic environment containing these anatomical structures. This will allow robust investigation of CT protocols in terms of radiation dose reduction, in addition to assessing the calcified plaques and coronary lumen.

## 5. Conclusions

In conclusion, this study has successfully created the high calcification for simulation of extensively calcified plaques in 3D-printed patient-specific coronary artery models. The coronary CT images reconstructed with the MIP and VR views clearly demonstrate these calcified plaques in different locations at the coronary branches. Measurement of plaque diameters is overestimated on both MIP and VR images, with measurement differences on the MIP images showing the smallest discrepancy, and the largest discrepancy on VR images. This preliminary study shows the feasibility of using 3D-printed coronary artery models for the investigation of optimal CT protocols to minimise the blooming artifacts resulting from high calcification in the coronary arteries.

## Figures and Tables

**Table 1:** Dimensional details of simulated calcified plaques in 3D-printed coronary artery models.

3D-Printed Models	No. of Plaques Inserted	Plaque Locations at Coronary Arteries with Dimensions: Diameter × Length (mm)			
		LM	LAD	LCx	RCA
Model 1	5	-	Plaque 1: 4 × 10 Plaque 2: 3 × 18	Plaque 3: 3 × 10	Plaque 4: 4 × 14 Plaque 5: 2 × 4.5
Model 2	2	-	-	Plaque 6: 1 × 10	Plaque 7: 1 × 17
Model 3	7	-	Plaque 8: 4 × 11 Plaque 9: 4 × 11 Plaque 10: 4 × 9	Plaque 11: 3 × 15	Plaque 12: 3 × 8 Plaque 13: 1 × 20 Plaque 14: 2 × 15
Model 4	2	Plaque 15: 3 × 8	-	-	Plaque 16: 4 × 13
Model 5	3	-	Plaque 17: 3 × 30	-	Plaque 18: 2 × 16 Plaque 19: 1 × 18
Model 6	3	-	Plaque 20: 1 × 30	-	Plaque 21: 4 × 16 Plaque 22: 3 × 16

LM—left main, LAD—left anterior descending, LCx—left circumflex, RCA—right coronary artery, - indicates no plaques inserted into these coronary arteries.

**Table 2:** Comparison of plaque diameter measurements between CT protocols using 0.5 mm and 1.0 mm slice thicknesses.

3D-Printed Models	Plaques at Left Main			Plaques at Left Anterior Descending			Plaques at Right Coronary Artery			*p*-Value
	Actual Diameter	0.5 mm Slice Thickness	1.0 mm Slice Thickness	Actual Diameter	0.5 mm Slice Thickness	1.0 mm Slice Thickness	Actual Diameter	0.5 mm Slice Thickness	1.0 mm Slice Thickness	
Model 1	-	-	-	4 and 3	4.06 ± 0.07 3.03 ± 0.03	4.07 ± 0.02 3.04 ± 0.01	4	4.16 ± 0.03	4.18 ± 0.04	0.65–0.89
Model 2	-	-	-	-	-	-	1	1.22 ± 0.03	1.15 ± 0.08	0.29
Model 3	-	-	-	4	4.10 ± 0.04	4.12 ± 0.10	3 and 2	3.18 ± 0.10 2.11 ± 0.01	3.17 ± 0.09 2.13 ± 0.06	0.69–0.97
Model 4	3	3.13 ± 0.04	3.15 ± 0.05	-	-	-	4	4.27 ± 0.09	4.22 ± 0.09	0.55–0.58
Model 5	-	-	-	3	3.04 ± 0.05	3.04 ± 0.01	2 and 1	2.22 ± 0.12 1.18 ± 0.11	2.21 ± 0.02 1.17 ± 0.05	0.83–0.92
Model 6	-	-	-	1	1.01 ± 0.09	1.06 ± 0.06	4 and 3	4.00 ± 0.02 3.06 ± 0.02	4.03 ± 0.05 3.05 ± 0.02	0.44–0.51

- indicates no plaques inserted into these coronary arteries.

**Table 3:** Diameter measurements of calcified plaques in 3D-printed coronary models when compared with the actual diameters.

3D-Printed Models	Plaques at Left Main				Plaques at Left Anterior Descending				Plaques at Left Circumflex				Plaques at Right Coronary Artery				*p*-Value
	AD	2D MIP	3D VR	3D VR *	AD	2D MIP	3D VR	3D VR *	AD	2D MIP	3D VR	3D VR *	AD	2D MIP	3D VR	3D VR *	
Model 1	-	-	-	-	4 and 3	4.07 ± 0.04 3.02 ± 0.04	4.29 ± 0.13 3.23 ± 0.09	4.21 ± 0.07 3.17 ± 0.03	3	3.02 ± 0.05	3.24 ± 0.01	3.13 ± 0.32	4 and 2	4.11 ± 0.02 2.04 ± 0.05	4.44 ± 0.10 2.35 ± 0.02	4.41 ± 0.09 2.30 ± 0.60	<0.05 0.25–0.78 *
Model 2	-	-	-	-	-		-	-	1	1.09 ± 0.08	1.61 ± 0.19	1.65 ± 0.13	1	1.12 ± 0.04	1.89 ± 0.08	1.84 ± 0.05	<0.05 0.50–0.78 *
Model 3	-	-	-	-	4, 4, and 4	4.12 ± 0.02 4.24 ± 0.01 4.18 ± 0.00	4.78 ± 0.04 4.71 ± 0.26 4.59 ± 0.16	4.65 ± 0.09 4.48 ± 0.14 4.35 ± 0.08	3	3.16 ± 0.02	3.62 ± 0.09	3.53 ± 0.06	3, 1 and 2	3.24 ± 0.05 1.23 ± 0.04 2.11 ± 0.03	3.77 ± 0.04 1.56 ± 0.08 2.58 ± 0.13	3.69 ± 0.04 1.44 ± 0.08 2.38 ± 0.09	<0.05 0.08–0.26 *
Model 4	3	3.16 ± 0.09	3.59 ± 0.11	3.75 ± 0.09	-		-	-	-	-	-	-	4	4.14 ± 0.07	4.71 ± 0.13	4.55 ± 0.08	<0.05
Model 5	-	-	-	-	3	3.04 ± 0.04	3.52 ± 0.16	3.26 ± 0.04	-		-	-	2 and 1	2.05 ± 0.01 1.13 ± 0.03	2.73 ± 0.09 1.50 ± 0.04	2.53 ± 0.11 1.42 ± 0.04	<0.05 0.06–0.09 *
Model 6	-	-	-	-	1	1.07 ± 0.01	1.39 ± 0.08	1.29 ± 0.10	-		-	-	4 and 3	4.04 ± 0.04 3.02 ± 0.01	4.67 ± 0.10 3.64 ± 0.11	4.43 ± 0.19 3.45 ± 0.16	<0.05 0.13–0.28 *

LM—left main, LAD—left anterior descending, LCx—left circumflex, RCA—right coronary artery. MIP—maximum-intensity projection, VR—volume rendering with wide window width to visualise both calcified plaques and coronary lumen, VR *—volume rendering with narrow window width to focus on calcified plaques. The *p*-value was less 0.5 when comparing measurements on 2D MIP with those on VR images in all of the plaques; the *p*-value (*) was more than 0.5 when comparing measurements on these two VR images in all of the plaques; AD—actual diameter, - indicates no plaques inserted into these coronary arteries.

**Table 4:** Length measurements of calcified plaques in 3D-printed coronary models when compared with the actual length.

3D-Printed Models	Plaques at Left Main				Plaques at Left Anterior Descending				Plaques at Left Circumflex				Plaques at Right Coronary Artery				*p*-Value
	AL	2D MIP	3D VR	3D VR *	AL	2D MIP	3D VR	3D VR *	AL	2D MIP	3D VR	3D VR *	AL	2D MIP	3D VR	3D VR *	
Model 1	-	-	-	-	10 and 18	10.33 ± 0.39 17.93 ± 0.35	10.34 ± 0.36 18.33 ± 0.15	10.14 ± 0.18 18.23 ± 0.05	10	9.70 ± 0.20	10.13 ± 0.30	9.99 ± 0.01	14 and 4.5	13.76 ± 0.30 4.42 ± 0.21	13.93 ± 0.46 4.56 ± 0.05	13.90 ± 0.20 4.52 ± 0.14	0.07–0.91
Model 2	-		-	-	-	-	-	-	10	9.55 ± 0.21	9.89 ± 0.31	9.88 ± 0.10	17	16.93 ± 0.32	16.90 ± 0.20	16.87 ± 0.32	0.07–0.88
Model 3	-	-	-	-	11, 11, and 9	10.76 ± 0.15 11.0 ± 0.17 9.12 ± 0.12	11.03 ± 0.15 11.03 ± 0.15 9.01 ± 0.15	10.63 ± 0.56 11.06 ± 0.40 9.0 ± 0.21	15	14.93 ± 0.25	14.96 ± 0.11	14.90 ± 0.17	8, 20, and 15	8.05 ± 0.03 19.73 ± 0.25 15.0 ± 0.10	8.07 ± 0.09 20.4 ± 0.36 15.13 ± 0.05	8.13 ± 0.17 19.96 ± 0.15 15.16 ± 0.05	0.06–0.93
Model 4	8	7.90 ± 0.05	7.96 ± 0.07	8.0 ± 0.04	-	-	-	-	-	-	-	-	13	12.93 ± 0.15	13.10 ± 0.20	13.06 ± 0.05	0.06–0.79
Model 5	-	-	-	-	30	30.06 ± 0.11	30.13 ± 0.23	30.10 ± 0.20	-	-	-	-	16 and 18	16.13 ± 0.11 17.96 ± 0.11	16.16 ± 0.11 18.06 ± 0.11	16.06 ± 0.05 17.96 ± 0.05	0.25–1.0
Model 6	-	-	-	-	30	29.86 ± 0.11	29.83 ± 0.32	29.80 ± 0.10	-	-	-	-	16 and 16	16.0 ± 0.00 16.06 ± 0.15	16.06 ± 0.05 16.13 ± 0.05	16.03 ± 0.05 16.06 ± 0.11	0.37–1.0

Abbreviations are the same as provided in Table 3. AL—actual length.

## Data Availability

The datasets used during the current study are not publicly available due to strict requirements set out by authorized investigators.
